# 

**Published:** 1898-03-12

**Authors:** 


**Notices of Births, Deaths, and Marriages** are inserted in this column at a charge of 2s. 6d. for an announcement not exceeding four lines.

### NOTICE TO CORRESPONDENTS.

All MS., Letters, Books for Review, and other matters intended for the Editor should be addressed The Editor, "The Hospital," The Hospital Building, 28 & 29, Southampton Street, Strand, W.C.

The Editor cannot undertake to return rejected MS., even when accompanied by stamped directed envelope.

**Notice to Advertisers and Subscribers.**

All Advertisements, Orders for Copies of The Hospital, and Business Communications should be addressed to **THE MANAGER, (Not to The Editor),** The Hospital, The Hospital Building, 28 & 29, Southampton Street, Strand, London, W.C.

The Cost of Subscription, post free, is as follows:—Per week, 2½d. per quarter, 2s. 8d.; per half-year, 5s. 3d.; per annum, 10s. 6d. Foreign:—Per annum, 15s. 2d.; payable, in all cases, in advance.

**NOTICE.—Vol. XXII. of "The Hospital" (April, 1897, to September, 1897), in handsome cloth binding, is now on sale at the Office of this Journal, price 7s. 6d. Cases for binding the Numbers contained in this Volume, 1s. 6d. Index to Vol. XXII., 2½d., post free. The Monthly Part for March is now ready. Price 6d., by post 8d.**

### BOOKS RECEIVED.

AIRD AND COGHILL, Glasgow.
"Old Age Pensions." By William Birkmire.

CASSELL AND CO.

"Cassell's Illustrated History of England." Part I.

F. A. DAVIS CO.
"Elements of Latin." By Geo. D. Crothers, A.M., M.D.
"Report of State Charities Aid Association, New York."
"Outlines of Rural Hygiene." By Harvey B. Bashore, M.D.

REBMAN PUBLISHING COMPANY.
"Pocket Formulary for the Treatment of Diseases of Children." By Ludwig Freyberger, M.D., M.R.C.P.

DIGBY, LONG, AND CO.

"Consumption." By B. Schwartzbach, M.D.

SWAN SONNENSCHEIN AND CO.
"Vaccination a Delusion." By Alfred Russel Wallace.

ARMY AND NAVY CO-OPERATIVE SOCIETY.
"Homes and Hospitals for the Benefit of Gentlewomen."

CHATTO AND WINDUS.
Herbert Fry's "Royal Guide to London Charities."

### The Student of Medicine.

### APPOINTMENTS.

L. M. Breton, L.R.C.P., M.R.C.S., L.S.A., appointed Medical Officer to the Southampton Dispensary and Provident Medical Institution. A. St. L. Burke, L.R.C.P., L.R.C.S.Irel., appointed Medical Officer for the Bushbury District of the Cannock Union. T. G. Dickson, L.R.C.P., L.R.C.S.Edin., appointed Medical Officer of Health to the Bredwardine Rural District Council. W. Gilbertson, B.A., M.R.C.S., L.R.C.P., appointed a Clinical Assistant to Out-patients at the Chelsea Hospital for Women. G. R. Green, L.R.C.P.Edin., M.R.C.S.Eng., appointed Medical Officer of Health for the Borough of Ripon. W. H. Gregory, M.D., C.M.Edin., appointed Public Vaccinator to the Second District of the Beverley Union. J. F. Holden, M.B., C.M.Edin., appointed Medical Officer for the Fourth District of the Preston Union. J. T. Jones, L.R.C.P., L.R.C.S.Edin., appointed Medical Officer of Health to the Llansilin Rural District Council. Dr. Evan Lloyd, appointed House Surgeon to the Flintshire Dispensary. A. A. Martin, M.B., B.S.Lond., M.R.C.S., L.R.C.P., appointed Assistant Medical Officer to the South-Eastern Fever Hospital, Hasfield Street, S.E. A. Miles, M.D., F.R.C.S.Edin., Surgeon to Leith Hospital, appointed Assistant Surgeon to the Edinburgh Royal Infirmary. H. C. Mooney, M.B., B.Ch., F.R.C.S.I., appointed Assistant Surgeon to Molesworth Street Branch of the Royal Victoria Eye and Ear Hospital, Dublin. J. I. Murray, M.D., F.R.C.S.Edin., appointed an Honorary Consulting Surgeon to the Sea Bathing Infirmary, Scarborough. A. W. Nuthall, M.R.C.S., L.R.C.P.Lond., appointed Second Assistant Medical Officer to the St. Marylebone Infirmary. P. H. Nutting, L.R.C.P.Lond., M.R.C.S.Eng., appointed Medical Officer for the Third District of the Malmesbury Union. J. H. Peet, L.R.C.P., L.R.C.S.Edin., appointed Medical Officer for the No. 8 District of the Basingstoke Union. W. Stephenson, M.R.C.S.Eng., appointed Medical Officer to the Beverley Rural District Council. S. T. Taylor, M.B.Lond., reappointed Medical Officer of Health to the Chingford Urban District Council. S. E. Tench, M.R.C.S., L.R.C.P., appointed a Clinical Assistant to Out-patients at the Chelsea Hospital for Women. H. L. Thurnell, M.A.Camb., M.R.C.S., L.R.C.P., appointed Medical Officer of Health for the Borough of Gravesend. O. B. Trumper, M.B., Ch.B. appointed Medical Officer of Health to the Market Rasen Urban District Council. Dr. Wynne, appointed Medical Officer for the No. 2 District of the Rye Union.

